# New York Journal of Medicine and the Collateral Sciences

**Published:** 1843-07

**Authors:** 


NEW YORK JOURNAL OF MEDICINE AND THE COLLATERAL SCIENCES.

We heard of this new Journal sometime since, but it is only very recently that we have seen the prospectus. It is to be under the editorial management of Dr. Samuel Forry, than whom no man in this country has a more growing reputation; the Journal could not be entrusted to better or more competent hands. It will appear every two months—which, we are inclined to think, is a happy medium between the monthly and quarterly. Messrs. J. & H. Langley are the proprietors.

New York formerly possessed one of the best medical periodicals ever published in the country—the New York Medical Repository; and to us, at this distance, no city in the Union would seem to offer more advantages for the new enterprise. We do not forget, whilst saying this, that there have been three unsuccessful attempts to sustain a medical periodical there, within the last few years. Dr. Swett's Journal (a very excellent one by the way) was suffered to go down; the Gazette, which succeeded it, shared the same fate; and the Lancet, which was the last, fairly run itself down. We hope a better fortune will await the new Journal. C.

